# Pancreatic cancer progression and mortality predicted by depression and anxiety: a systematic review and meta-analysis protocol

**DOI:** 10.3389/fpsyt.2023.1266502

**Published:** 2024-01-11

**Authors:** Ruoqi Zhang, Jing Wang, Peitong Zhang, Zheng Zhang, Rui Miao

**Affiliations:** ^1^Department of Oncology, Guang'anmen Hospital, China Academy of Chinese Medical Sciences, Beijing, China; ^2^Graduate College, Beijing University of Chinese Medicine, Beijing, China

**Keywords:** Pancreatic Neoplasms, depression, anxiety, prognosis, mortality rate

## Abstract

Although the relationship between psychological factors and pancreatic cancer outcomes has been widely discussed, controversy remains. We will for the first time systematically summarize the literature to explore the correlation of anxiety and depression to the prognosis of patients with pancreatic cancer. The findings will fill existing research gaps, informing healthcare providers about better psychological care and medical treatment. The following databases will be retrieved from their inception to July 2023: Cochrane Library, MEDLINE (PubMed), Web of Science, EMBASE, and four Chinese databases (Chinese National Knowledge Infrastructure, Wanfang Database, Chinese Biomedical Literature Database, and Chongqing VIP Chinese Science and Technology Periodical Database). The World Health Organization Clinical Trials Registry, Chinese Clinical Registry, and ClinicalTrials.gov will be searched to identify other related studies. A manual search will be performed to identify missing eligible studies based on the reference list of selected articles. The search will focus on studies published in Chinese or English. To assess the risk of bias in the selected articles, Newcastle-Ottawa Quality Assessment Scale (NOS) will be used for the cohort study. Funnel plots and Egger's test will be used to assess whether publication bias exists. Moreover, the Grading of Recommendations Assessment Development and Evaluation (GRADE) will be utilized to analyze the credibility of the results from selected articles. Two independent evaluators will implement the study selection and data extraction, as well as evaluate the risk of bias and evidence quality. Data will be analyzed using Stata 16.0.

**Trial registration:** PROSPERO registration number is CRD42022366232.

## 1 Introduction

Pancreatic cancer is a highly malignant cancer with mortality approaching its incidence rate. As one of the leading causes of cancer death worldwide, the risk of pancreatic cancer is higher among men in and high-income regions and may increase with population aging (1). The survival rate has improved in recent years, owing to palliative therapies and adjuvant treatment development (2). The search for more accurate histological and serological markers provides multiple benefits for the clinical management of patients with pancreatic cancer, particularly in terms of diagnosis or prognosis (3). However, patients with pancreatic cancer are often diagnosed at advanced stages or distant metastasis due to insidious early symptoms and the aggressive nature of the cancer, which leads to the 5-year survival rate remaining poor (4, 5). In addition, several risk factors of cancer-related deaths are closely linked to rapid progression and high mortality. Therefore, identifying the factors affecting cancer-specific mortality is crucial to improve the outcomes and slow disease progression.

The rapid progression of pancreatic cancer is facilitated by certain risk factors. For example, studies have found that patients with chronic pancreatitis are eight times more likely to develop pancreatic cancer than the general population in 5 years after diagnosis (6, 7). Furthermore, lifestyle and inherited risk factors, such as smoking, obesity, diabetes, and genetic changes, should also be considered (8). Meanwhile, with progress in comprehensive treatment, psychological factors are crucial to the occurrence and progression of pancreatic cancer. There is increasing evidence that cancer and depression co-exist through complex biological mechanisms, such as psychological stress, circadian rhythm disturbances, inflammatory responses, intestinal dysbiosis, and neurotransmission abnormalities (9).

Psychological factors play an important role in pancreatic cancer development compared with other solid tumors. Patients with pancreatic cancer often endure unpleasant side effects, lower quality of life, and heavy financial difficulties (10–13). Consequently, they usually experience emotional anguish and psychological issues. Research has verified that patients with pancreatic cancer suffer more extreme mental disturbances than those with other neoplasms (14, 15). The most common emotional disorders, depression and anxiety, may affect quality of life, motivation for treatment, and cancer outcomes, and even become the major cause of death (16). Despite the extensive discussion on how psychological factors affect pancreatic cancer-related mortality, the findings remain controversial. Seoud et al. (17) found that patients with depression after a diagnosis of pancreatic cancer has a higher risk of all-cause mortality compared with patients with pancreatic cancer alone.

It has also been found that patients with both pancreatic cancer and psychiatric disorders experience a higher risk of death from all causes or cancer, with anxiety and depression being the most common psychiatric disorders (18). Two studies have reported that depression was linked to a higher mortality rate and high heterogeneity for mixed tumor types (19, 20). Furthermore, patients with pancreatic cancer who experience depression may be more likely to attempt or complete suicide, which may lead to poorer all-cause mortality (21). Regarding the relationship between depression relief and survival benefits, two studies reported that lower depression levels predicted better survival outcomes in patients with pancreatic cancer (22, 23). In contrast, another study found that depression did not affect survival outcome (24). Additionally, whether anxiety predicts pancreatic cancer progression remains unclear. Four studies have found that anxiety was related to poorer survival rates (22, 25–27), two of which did not limit the type of cancer (26, 27). Moreover, a previous meta-analysis showed that anxiety was a risk factor for both cancer-specific and all-cause mortality among cancer patients (28). However, Walker et al. found that anxiety was related to better survival in female cancer patients (29). In another study, anxiety was not significantly related to increased morbidity or mortality risks in patients after organ transplantation (30). It is clear that existing studies have not drawn definite conclusions about the impact of depression and anxiety on the progression and outcome of patients with pancreatic cancer. Therefore, it is important to identify these through a systematic review and meta-analysis.

Our study is designed to assess how depression and anxiety affect the progression and mortality of patients with pancreatic cancer. We will further explore (a) whether depression and anxiety are diagnosed using standard diagnostic criteria or self-report scales, (b) the time of assessment of anxiety and depression, (c) the age of patients, (d) follow-up duration, and (e) whether the use of sufficient data can determine whether severity levels of anxiety and depression have different effects on pancreatic cancer progression and mortality.

## 2 Methods

### 2.1 Study criteria for this review

#### 2.1.1 Study designs and characteristics

This study is designed as a systematic review and meta-analysis protocol. We have registered in the PROSPERO International Prospective Register of Systematic Reviews (CRD42022366232) (https://www.crd.york.ac.uk/PROSPERO) and reported following the guidelines outlined in the Preferred Reporting Items for Systematic Review and Meta-Analysis Protocols (PRISMA-P) statement (31).

Inclusion will be granted to cohort studies, regardless of whether they are prospective or retrospective. Cross-sectional studies, case reports, reviews, abstracts, comments, and letters will be excluded.

### 2.2 Participants

Participants (over the age of 18) with pancreatic cancer diagnosed by histology or pathology, with or without depression or anxiety, will be included. Participants with malignancies other than pancreatic cancer will be excluded to avoid confounding factors. There will be no restrictions on sex or race.

### 2.3 Exposure

The exposure factor of interest is depression or anxiety diagnosed using standard diagnostic criteria or self-report scales.

### 2.4 Comparators

Patients without depression or anxiety will be assigned to the comparator group.

### 2.5 Outcome measures

The outcomes of selected studies should contain at least one of the following items:

a. progression-free survival (up to the point of death or progression of pancreatic cancer).
b. all-cause mortality (mortality due to any cause).
c. pancreatic cancer-specific mortality (death resulting from pancreatic cancer).

### 2.6 Search methods

#### 2.6.1 Searching electronic databases

The following databases will be retrieved from their inception to July 2023: Cochrane Library, MEDLINE (PubMed), Web of Science, EMBASE, and four Chinese databases (Chinese National Knowledge Infrastructure, Wanfang Database, Chinese Biomedical Literature Database, and Chongqing VIP Chinese Science and Technology Periodical Database). The search process will focus only on studies published in Chinese or English.

#### 2.6.2 Searching other resources

The WHO Clinical Trials Registry, Chinese Clinical Registry, and ClinicalTrials.gov will be searched to identify other related studies. Manual searches will be performed to identify missing eligible studies based on the reference lists of selected articles.

#### 2.6.3. Search strategies

We will search for MeSH terms, titles/abstracts, and publication types including emotional disorder (depression OR depressive disorder OR anxiety OR psychological distress OR mental disorder), pancreatic cancer (Pancreatic Neoplasms OR Pancreatic Tumor OR Pancreatic Cancer OR Pancreatic Carcinoma), outcome (survival OR mortality OR metastasis OR progression), and research design (cohort study OR prospective study OR longitudinal study OR follow-up).

The extensive search strategy for MEDLINE is depicted in Table 1, which will be revised for other databases.

**Table 1:** MEDLINE (by PubMed) search strategy.

Depression [MeSH] OR depression [ti/ab] OR depressive disorder [ti/ab] OR anxiety [MeSH] OR anxiety [ti/ab] OR psychological distress [ti/ab] OR mental health condition [tiab]
Pancreatic Neoplasms [MeSH] OR Pancreatic Neoplasms [ti/ab] OR Pancreatic Tumor [ti/ab] OR Pancreatic Cancer [ti/ab] OR Pancreatic Carcinoma [ti/ab]
Survival [ti/ab] OR mortality [ti/ab] OR metastasis [ti/ab] OR progression [ti/ab]
Cohort studies [MeSH] OR cohort studies [ti/ab] prospective studies [ti/ab] OR longitudinal studies [ti/ab] OR follow-up [ti/ab]
#1 and #2 and #3 and #4

## 3 Data collection and analysis

### 3.1 Study selection

**Figure 1:**
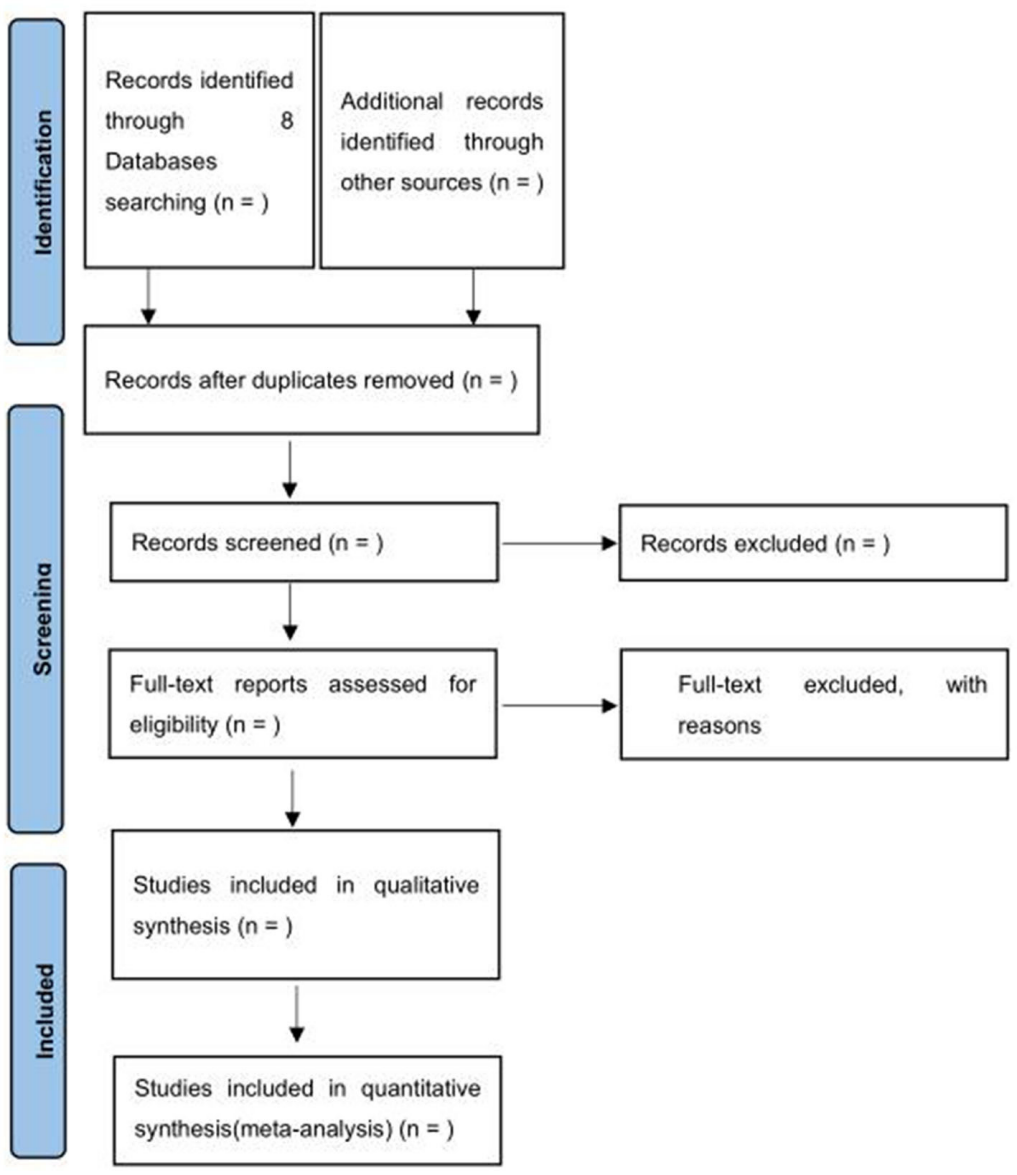
Preferred reporting items for systematic and review and meta-analysis (PRISMA) 2020 flow diagram.

Two experienced evaluators (Z-RQ and WJ) will assess the eligibility of each selected study independently based on the title, keywords, and abstract, if applicable. They will then read the full text to further judge whether it meets the preset inclusion criteria. The reasons for excluding the studies will be recorded. Any disagreements over the inclusion of studies will be resolved by a third reviewer with experience (Z-PT), and 10% of the included papers will be verified randomly for accuracy. We will get in touch with the authors for further information to confirm the eligibility if necessary. When multiple studies report similar results, preference will be given to the latest and/or complete study for data aggregation. The research screening procedure is described in Figure 1.

### 3.2 Data extraction and management

Two independent researchers (Z-RQ and WJ) will extract and gather the following information according to a predetermined form:

a. Studies (first author name, publication year, country, sample capacity, follow-up period, and quality of evidence).
b. Patients (age, race, gender, and stage of PC).
c. Exposure (time of depression/anxiety assessment, depression/anxiety measurement, and severity of depression/anxiety).
d. Outcome evaluation and adjusted major confounders.

### 3.3 Evaluation of the risk of bias

We will utilize the NOS (32) for cohort studies to evaluate the risk of bias of the selected studies. This scale evaluates the risk of bias through a rating system. The following three aspects of the included studies will be assessed: selection, comparability, and outcome evaluation or follow-up duration. Two evaluators will classify the risk of bias into three levels: low risk (≥7 score), fair (4–6 score), and high risk (< 4 score). A third reviewer (Z-PT) will be consulted if there are any disagreements between the two reviewers. Moreover, Egger's test and funnel plots will be used to assess whether the publication bias exists (33). A statistical publication bias will be taken into account when the *P*-value is < 0.10 on Egger's test.

### 3.4 Quality evaluation

The reliability of the evidence from selected articles will be assessed using GRADE (34). The GRADE starts from a low level when applied to observational data and can then be upgraded or downgraded. There are three situations where confidence increases (dose-response gradient, large effect size, and the reduced effect caused by confounders) and five domains with reduced confidence (risk of bias, inconsistency, inaccuracy, indirectness, and publication bias) (35).

### 3.5 Outcome measurement

We will assess the outcome data using hazard ratios (HR) and 95% confidence intervals (95% CI) for each study. The relative risk (RR) will be considered approximately equal to HR, if the HR is not reported.

### 3.6 Dealing with missing data

The corresponding author of the study will be contacted (with up to three emails) to request the missing information. If the RR or HR are not reported or available after contacting the authors, these data will not be included in the analysis.

### 3.7 Heterogeneity evaluation

We will employ Cochran's *Q*-test and *I*^2^ statistics to assess the heterogeneity of the included studies. The *I*^2^ statistics values of 25, 50, and 75% indicate low, medium, and high heterogeneity, respectively. The *Q*-test with *P* > 0.10 and *I*^2^ < 50% representing the statistical heterogeneity among the results of the included studies is acceptable. Otherwise, subgroup and sensitivity analyses are necessary to analyze the potential causes of heterogeneity.

### 3.8 Data synthesis

Statistical analyses will be performed using Stata 16.0. A fixed-effects model will be used to combine the data with acceptable statistical heterogeneity. Otherwise, we prefer the random-effects model and subgroup and sensitivity analyses to explore the sources of heterogeneity. However, if heterogeneity is significant, relevant explanations will be presented descriptively.

### 3.9 Subgroup analysis and heterogeneity investigation

A subgroup analysis will be performed to explore the source of heterogeneity according to the following potential factors:

a. average age ( ≤ 60 vs. >60 years)
b. follow-up period (< 3 vs. ≥3 years)
c. time for evaluation of mental disorder (before vs. after carcinoma diagnosis)
d. measurement of emotional state (clinical diagnosis vs. symptom scale)
e. severity of anxiety and depression (mild, moderate, or severe)

### 3.10 Sensitivity analysis

A sensitivity analysis will be conducted to validate the stability of the results. We will remove one study each time while reanalyzing the remaining studies. The stability of the integrated results will be determined by comparing the results before and after.

### 3.11 Patient engagement

Neither the patients nor the general public will be engaged in the creation, implementation, or reporting of this study.

### 3.12 Ethics and dissemination

Our study has no obvious ethical issues, as we will use data extracted from publicly accessible databases. The results of this study are expected to be published in an open, peer-reviewed journal to encourage future research.

## 4 Discussion

The high mortality rate and complex accompanying symptoms of pancreatic cancer patients lead to poor quality of life. Meanwhile, worsening mental problems can become a serious and widespread difficulty. If psychological problems are not well-managed, they may accelerate the progression of pancreatic cancer. Although the theoretical mechanism of psychological problems that accelerate pancreatic cancer progression has been widely discussed, clinical validation remains insufficient. Therefore, a systematic review is necessary that acts as a higher-level evidence-based tool.

A previous study indicated that depression and anxiety are linked to an increased risk of both cancer-specific and all-cause mortality in cancer patients, but their impact on cancer outcomes may vary between different cancer types (28). To date, only one systematic review has been performed to explore how depression and anxiety affect survival and treatment compliance (chemotherapy or pancreatectomy) in patients with pancreatic cancer (22). Notably, researchers matched patients without a cancer diagnosis to patients with pancreatic cancer according to age and gender, rather than matching patients based on whether they had depression/anxiety. Moreover, the diagnoses of psychiatric disorders were identified according to electronic medical records (EMR), which might underestimate its true incidence.

Global research has shown that the impact of psychological factors on pancreatic cancer-related death remains controversial. The inconsistent selection of participants and confounding factors between studies may account for this situation, making direct comparisons more difficult.

Consequently, a thorough and rigorous meta-analysis is necessary to investigate the effects of depression and anxiety on pancreatic cancer outcomes. We aim to comprehensively analyze the relationship between pancreatic cancer progression, anxiety, and depression. To the best of our knowledge, this study will be the first to explore this question.

Forest plots will be used to estimate progression-free survival, all-cause, and pancreatic cancer-specific mortality by overall pooled estimates based on the research included. Identifying the risk factors influencing progression-free survival and pancreatic cancer-related deaths is critical for improving prognosis and outcomes. Moreover, a leave-one-out sensitivity analysis will be implemented to validate the stability of results by comparing the prior and subsequent outcomes. Additionally, we will explore the study heterogeneity based on the age of the patients, follow-up duration, evaluation time of psychological disorders, and mental state measurement using subgroup analysis. If sufficient data are available, we will explore the impact of the severity of anxiety and depression on pancreatic cancer outcomes in subgroup analyses. These factors will be well-considered in the assessment of psychological problems and progression of pancreatic cancer.

There are several limitations to this study that should be mentioned. We are limiting the languages to English and Chinese when searching the literature. In addition, we do not limit the histological subtypes and tumor stages; thus, the risk of heterogeneity will inevitable.

Here, we present a protocol for systematic review and meta-analysis to assess the impact of depression and anxiety on the progression and mortality of patients with pancreatic cancer. The findings will fill existing research gaps, informing healthcare providers about better psychological care and medical treatment in patients with pancreatic cancer. We will publish the results in a peer-reviewed journal and other multiple digital platforms.

### 4.1 Strengths and limitations

Regarding strengths, (a) to our knowledge, no previous systematic review has explored the effects of depression and anxiety on pancreatic cancer progression and mortality, and (b) two independent reviewers will be responsible for selecting studies, extracting data, evaluating bias, and assessing quality. Regarding limitations, (c) the search will be limited to English and Chinese databases, (d) and no restrictions will be placed on histological subtypes or tumor stages, which will inevitably increase the risk of heterogeneity.

## Ethics statement

This study follows the guidelines from the Preferred Reporting Items for Systematic Review and Meta-Analysis Protocols (PRISMA-P) checklist. It has no obvious ethical issues, as we will use data extracted from publicly accessible databases. The results are expected to be published in an open, peer-reviewed journal to encourage future research.

## Author contributions

RZ: Methodology, Resources, Visualization, Writing—original draft. JW: Methodology, Writing—review & editing. PZ: Methodology, Supervision, Writing—review & editing. ZZ: Methodology, Writing—review & editing. RM: Methodology, Writing—review & editing.
